# Synthesis, structural characterizations, in vitro biological evaluation and computational investigations of pyrazole derivatives as potential antidiabetic and antioxidant agents

**DOI:** 10.1038/s41598-024-51290-6

**Published:** 2024-01-15

**Authors:** Salma Mortada, Khalid Karrouchi, El Hadki Hamza, Afaf Oulmidi, Mashooq Ahamd Bhat, Hassane Mamad, Youssra Aalilou, Smaail Radi, M’hammed Ansar, Azlarab Masrar, My El Abbes Faouzi

**Affiliations:** 1 Laboratory of Pharmacology and Toxicology, Biopharmaceutical and Toxicological Analysis Research Team, Faculty of Medicine and Pharmacy, Mohammed V University, Rabat, Morocco; 2 Laboratory of Analytical Chemistry and Bromatology, Team of Formulation and Quality Control of Health Products, Faculty of Medicine and Pharmacy, Mohammed V University in Rabat, Rabat, Morocco; 3 CERNE2D: Laboratory of Spectroscopy, Molecular Modelling, Materials, Nanomaterials, Water and Enviroment (LS3MN2E), Faculty of Sciences, Mohammed V University, Rabat, Morocco; 4 Institute of Condensed Matter and Nanosciences, Molecular Chemistry, Materials and Catalysis (IMCN/MOST), Université Catholique de Louvain, 1348 Louvain-la-Neuve, Belgium; 5 Department of Pharmaceutical Chemistry, College of Pharmacy, King Saud University, 11451 Riyadh, Saudi Arabia; 6 Central Laboratory of Hematology, Ibn Sina Hospital, Faculty of Medicine and Pharmacy, Mohammed V University, Rabat, Morocco; 7 Laboratoire de Chimie Appliquée et Environnement (LCAE), Faculté des Sciences, Université Mohammed I, 60000 Oujda, Morocco; 8 Laboratory of Medicinal Chemistry, Faculty of Medicine and Pharmacy, Mohammed V University, Rabat, Morocco

**Keywords:** Drug discovery, Chemistry

## Abstract

In this study, a two pyrazole derivatives; 2-(5-methyl-1H-pyrazole-3-carbonyl)-N-phenylhydrazine-1-carboxamide (**Pyz-1**) and 4-amino-5-(5-methyl-1H-pyrazol-3-yl)-4H-1,2,4-triazole-3-thiol (**Pyz-2**) were synthesized and characterized by ^13^C-NMR, ^1^H-NMR, FT-IR, and mass spectrometry. A complete molecular structures optimization, electronic and thermodynamic properties of **Pyz-1** and **Pyz-2** in gas phase and aqueous solution were predicted by using hybrid B3LYP method with the 6-311++G** basis sets. **Pyz-1** and **Pyz-2** were evaluated in vitro for their anti-diabetic, antioxidant and xanthine oxidase inhibition activities. For anti-diabetic activity, **Pyz-1** and **Pyz-2** showed a potent α-glucosidase and α-amylase inhibition with IC_50_ values of 75.62 ± 0.56, 95.85 ± 0.92 and 119.3 ± 0.75, 120.2 ± 0.68 µM, respectively, compared to Acarbose (IC_50(α-glucosidase)_ = 72.58 ± 0.68 µM, IC_50(α-amylase)_ = 115.6 ± 0.574 µM). In xanthine oxidase assay, **Pyz-1** and **Pyz-2** exhibited remarkable inhibitory ability with IC_50_ values 24.32 ± 0.78 and 10.75 ± 0.54 µM, respectively. The result of antioxidant activities showed that the title compounds have considerable antioxidant and radical scavenger abilities. In addition, molecular docking simulation was used to determine the binding modes and energies between the title compounds and α-glucosidase and α-amylase enzymes.

## Introduction

Type 2 diabetes mellitus (T2DM) is characterized by high blood glucose levels, which can lead to major complications such as cardiovascular disease, neuropathy, retinopathy and kidney disease^1^. The disease affects 4.9 million people worldwide, with 90% of diabetes cases attributable to T2DM. It is one of the most common public health problems^2–5^. Controlling postprandial hyperglycemia in T2DM by inhibiting dietary carbohydrate digestion (α-amylase and α-glucosidase) is considered one of the most effective therapeutic approaches for lowering blood glucose levels. Thus, α-amylase and α-glucosidase are two enzymes essential for breaking down and degrading dietary carbohydrates such as starch in the digestive tract into simple monosaccharides, in particular, glucose, which passes into the bloodstream after absorption. Consequently, enzymatic inhibition of α-amylase and α-glucosidase can suppress carbohydrate digestion, delay glucose absorption and, consequently, lead to a reduction in blood glucose levels^6–8^. On the other hand, several chronic diseases such as T2DM have been related to oxidative stress which entails the production of reactive oxygen species (ROS) such as the hydroxyl radical (OH^–^), hydrogen peroxide (H_2_O_2_) and superoxide anion radical (O_2_^–^)^9^. On other hand, the increase of malondialdehyde (MDA) concentration by lipid peroxidation in pancreatic tissue of diabetic animal models confirmed the role of ROS in the pathogenesis and progression of diabetes. Thus, in vivo animal studies have shown that potent inhibition of oxidative stress by specific anti-oxidants under experimental diabetic conditions has shown preventive effects on the progression of diabetic complications^10,11^. Therefore, it would be very beneficial to develop new compounds with both anti-diabetic and antioxidant activities to minimize adverse effects.

Indeed, pyrazole constitute a large class of heterocycles which can be explored for the development of new drug substances. An extensive study of this class has demonstrated that pyrazole can be present in various known drugs of different classes with different therapeutic activities^12,13^. In the last few years, pyrazole chemistry and its derivatives have attracted considerable interest due to their broad spectrum of biological activities, such as antidiabetic^14^, antibacterial^15,16^, antioxidant and analgesic^17^, anticancer^18^, antiviral^19^ and antituberculosis agents^20^. Considering the biological and pharmacological properties of these derivatives, it is essential to study their electronic and structural properties to know the influence of the different groups on their biological properties^21,22^. For this purpose, computational approaches are known for their efficiency and accuracy when it comes to predict and understand the properties of novel synthesized organic molecules^23^. In continuation of our efforts to develop new potential anti-diabetic and antioxidant agents^13,19,20^, we report herein the synthesis and characterization of two pyrazole derivatives **Pyz-1** and **Pyz-2** as new potent antiadiabetic and antioxidant agents. The molecular geometry and electronic characters of these molecules were explored by using DFT calculations. Thus, the title compounds were evaluated in vitro for their α-glucosidase and α-amylase enzymes inhibitory. Antioxidant activities of **Pyz-1** and **Pyz-2** were determined by DPPH, ABTS, FRAP, H_2_O_2_ radical scavenging activity assays and Xanthine Oxidase (XO) inhibition assay. In addition, molecular docking and ADMET studies were also performed.

## Experimental

### Reagents and instruments

Chemical reagents (Methanol, Ethanol, Phenyl isocyante, Potassium hydroxide, hydrazine hydrate, Carbon disulfide and Hydrochloric acid) were purchased from Fluka, Sigma and Aldrich chemicals. Melting points were measured using a Buchi B-545 digital capillary melting point apparatus. TLC with silica gel 60 F254 were used to check the reactions. The IR spectra were recorded by using Perkin-Elmer VERTEX 70 FT-IR spectrometer covering field 400–4.000 cm^−1^. The ^1^H NMR and ^13^C NMR spectra were recorded using JNM-ECZ500R/S1 FT NMR SYSTEM (JEOL) (^1^H 500 MHz/^13^C 125 MHz) spectrometer by using dry deuterated DMSO as solvent. The Mass spectra were obtained using an API 3200 LC/MS/MS spectrometer.

### Chemistry

#### Procedure for the synthesis of 2-(5-methyl-1H-pyrazole-3-carbonyl)-N-phenylhydrazine-1-carboxamide (Pyz-1)

5-methyl-1H-pyrazole-3-carbohydrazide (**1**) was synthesized according the previously reported method^14,17^. Phenyl isocyanate (0.5 g, 4.2 mmol) was added dropwise to a solution of 5-methyl-*1H*-pyrazole-3-carbohydrazide (**1**) (0.5 g, 3.75 mmol) in methanol (20 ml), and the mixture was refluxed for 8 h. The solid was filtered and recrystallized from ethanol to afford pure product (**Pyz-1**). White solid; Yield = 81%; m.p = 252–254 °C; FT-IR (ATR, cm^−1^): 3129–3295 (NH), 2934–3000 (CH), 1703, 1650 (C = O), 1602 (C = N); ^1^H NMR: (500 MHz, DMSO-*d*_*6*_, δ(ppm)): 2.23 (3H, s, CH_3_), 6.42 (1H, s, CH-pyrazole), 6.89–7.43 (m, 5H, H-Ar), 8.04 (s, 1H, NH), 8.73 (s, 1H, NH), 9.75 (s, 1H, NH), 12.99 (1H, s, NH-pz); ^13^C NMR: (125 MHz, DMSO-*d*_*6*_, δ (ppm)): 10.82, 105.06, 118.87, 122.31, 129.18, 140.17, 140.27, 145.89, 156.04, 162.61. ESI-HRMS: *m/z* calcd. for C_12_H_13_N_5_O_2_ [M-H]^–^: 258.1102, found 258.0986.

#### Procedure for the synthesis of 4-amino-5-(5-methyl-1H-pyrazol-3-yl)-4H-1,2,4-triazole-3-thiol (Pyz-2)

To an ice cooled solution of 5-methyl-1H-pyrazole-3-carbohydrazide (**1**) (0.5 g, 3.75 mmol) and KOH (0.2 g, 3.75 mmol) in absolute ethanol (15 mL), was added CS_2_ (0.65 ml, 10.71 mmol) dropwise, and the mixture was stirred at room temperature for 12 h. The separated solid was filtered and washed with ethanol. The obtained pyrazole-potassium salt was used in the next reaction without further purification. To a solution of the pyrazole carbodthionate (0.5 g, 1.97 mmol) in ethanol (10 ml) was added hydrazine hydrate (1 ml) and the mixture was refluxed for 8 h. The completion of the reaction was monitored by TLC. The reaction mixture was diluted with cold water and neutralized with concentrated HCl. The solid was filtered and recrystallized from ethanol to afford pure product (**Pyz-2**). White solid; Yield = 67%; m.p = 216–218 °C; FT-IR (ATR, cm^−1^): 3144–3367 (NH, NH_2_), 2732 (SH); ^1^H NMR: (500 MHz, DMSO-*d*_*6*_, δ(ppm)): 2.25 (3H, s, CH_3_), 5.89 (2H, s, NH_2_), 6.60 (1H, s, CHpyrazole), 13.09 (1H, s, NH-pz), 13.66 (1H, s, SH); ^13^C NMR: (125 MHz, DMSO-*d*_*6*_, δ (ppm)): 10.71, 105.08, 138.78, 139.97, 145.46, 165.17; ESI-HRMS: *m/z* calcd. for C_6_H_8_N_6_S [M + H]^+^: 197.0545, found 197.0586.

### Computational details

Two series of calculations were conducted in an attempt to deduce and interpret the experimental data. First, The DFT computations were carried out in order to gather information regarding the reactivity of the synthesized compounds. Then, a molecular docking simulation was used to determine the binding modes and energies between the compounds (**Pyz-1** and **Pyz-2**) and α-glucosidase and α-amylase enzymes. A complete molecular structures optimization was performed using the B3LYP hybrid functional^24,25^ in conjunction with the 6-311G +  + (d,p)^26–30^ basis set in order to determine the respective total energy and the most stable gas-phase geometry. Throughout the geometry optimization, no symmetry constraints were imposed. The absence of imaginary frequencies in the calculation of the vibrational modes indicates that these structures correspond to real local minima on the potential energy surface. The effects of water as a solvent (ε = 46.7) were examined using a self-consistent reaction field (SCRF)^31^ based on the polarizable continuum model (PCM) of the Thomasi group^32,33^ via a single-point calculation of the optimized geometries performed in the phase gas. Molecular electrostatic potential (MEP) surfaces were obtained using the Multiwfn program^34^ at the same level of theory. The obtained results were visualized using the VMD 1.9 software^35^. Gaussian NBO Version 3.1^36–38^ was used to perform the natural bond orbital (NBO) calculations. To analyze and evaluate the tendency of the drug for charge transfer processes and drug-receptor interactions^39^, several theoretical reactivity parameters^40^ based on the conceptual density functional theory (CDFT)^41^. All calculations were performed using the Gaussian09 suite of program^42^ and GaussView graphical interface^43^.

### Molecular docking

To investigate possible binding modes of selected chemical entities, a docking simulation employing the AutodockVina v1.5.6^44^ was carried out targeting the α-glucosidase and α-amylase enzyme active sites^45^. **Pyz-1**, **Pyz-2** and Acarbose structures have been designed by using ChemDraw softaware package and were optimized using the molecular building module implemented in ChemDraw. Thus, crystal structure of α-glucosidase (PDB Id: 3A4A) and α-amylase (PDB Id: 2GJP) were downloaded from the PDB database (https://www.rcsb.org/pdb). Potential binding sites of α-amylase and α-glucosidase were identified using Discovery Studio software^45^, based on the receptor structure without prior introduction of the ligand. The preparation of the binding site geometry was performed using AutoGrid, employing a grid with multiple points defined by coordinates (x, y, z) = (20, 20, 20), with a grid spacing of 0.375 Å. The different interactions within the protein–ligand complexes were analyzed using the Discovery 2016 Studio Visualizer^45^ and PyMol molecular graphing system^46^**.** Pharmacokinetic and pharmacodynamic parameters of **Pyz-1** and **Pyz-2** were computed by means of the ADMETLab2.0 server^47^.

### Biology

#### α-Glucosidase and α-amylase inhibitory assays

The α-glucosidase and α-amylase inhibitory activities of compounds **Pyz-1** and **Pyz-2** were determined using the method described in our previous works^48,49^.

#### Antioxidant activities

The antioxidant activities of the title compound were determined in vitro by DPPH, ABTS, FRAP and hydrogen peroxide activity (H_2_O_2_) methods according to the procedures described in our previous works^49^.

#### Xanthine oxidase inhibition assay (XO)

The xanthine oxidase inhibitory activity of **Pyz-1** and **Pyz-2** was performed following the method described by Kostić et al.^50^, with minor modifications. The samples were prepared at series of concentrations. 1.95 ml of 5 mM phosphate buffer pH 7.5 and 50 µl of enzyme solution was added to the mixtures. After pre-incubation at 25 °C for 15 min. 1 ml of substrate was added. After 30 min add 0.5 ml of HCl to stop the reaction the absorbance was determined spectrophotometrically at 295 nm associated with uric acid formation.

### Statistical analysis

Statistical analyze was performed using *GraphPad Prism8* program. One-way ANOVA was used to determine the significant difference. Quantitative data were presented as mean ± standard error of the mean (SEM) and *p* < *0.0001* was considered statistically highly significant.

## Results and discussion

### Chemistry

**Scheme 1:**
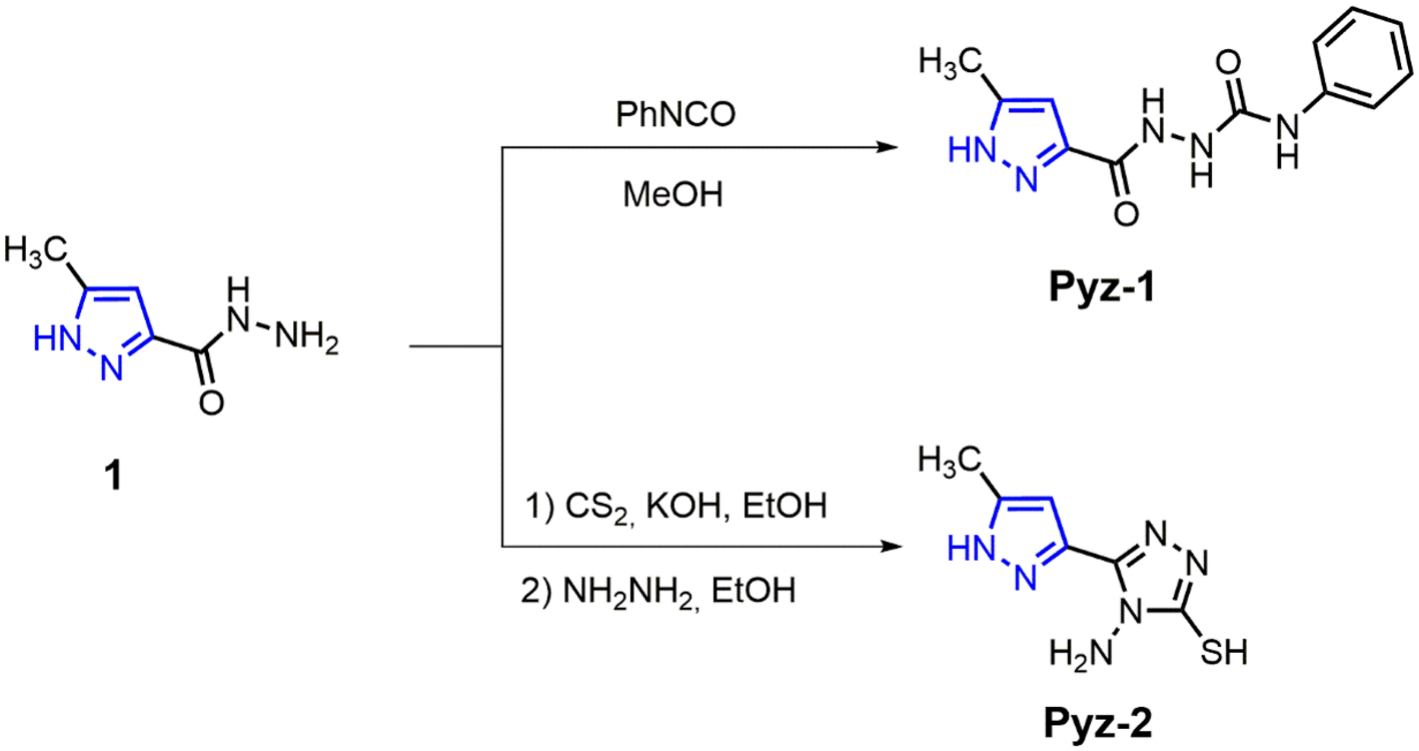
Synthetic route for preparation of compounds Pyz-1 and Pyz-2.

First, the synthesis protocol of **Pyz-1** and **Pyz-2** compounds was performed according to Scheme 1. The starting hydrazide acid (**1**) was synthesized according the previously reported method^49^. The compound (**1**) was treated with CS_2_ in ethanol in the presence of KOH as base at room temperature to afford the intermediate potassium salt in quantitative yield. Thus, treatment of this intermediate with hydrazine hydrate under ethanol reflux afforded a pure product (**Pyz-1**) in good yield. Subsequently, 2-(5-methyl-1H-pyrazole-3-carbonyl)-N-phenylhydrazine-1-carbothioamide (**Pyz-2**) was prepared in excellent yield by condensation of pyrazole hydrazide (**1**) and phenyl isocyanate under reflux of methanol.

The molecular structures of **Pyz-1** and **Pyz-2** were confirmed by using ^1^H-NMR, ^13^C-NMR and ESI–MS spectrometry. The ^1^H NMR spectrum of **Pyz-1** shows a singlet at 2.18 ppm characteristic of methyl group, a singlet at 6.42 ppm due to the proton in position 3 of the pyrazole ring. Aromatic protons of phenyl group resonate as multiplet at 6.92–7.44 ppm. The NH protons of carboxamide group appear as three singlets at 8.02, 8.72 and 9.74 ppm. The singlet at 12.99 ppm due to the NH group of pyrazole. The ^13^C NMR spectrum of **Pyz-1**, shows a signal at 10.75 ppm characteristic to carbon of the CH_3_ group, a signal at 105.97 ppm characteristic to the tertiary carbon of pyrazole, followed by the carbons of the aromatic part which appear between 118.78 and 140.9 ppm. The two quaternary carbons of pyrazole resonate at 140.19 and 145.81 ppm. The two signals observed at 155.94 and 162.51 ppm are attributed to the two C = O groups.

The ^1^H NMR spectrum of **Pyz-2** shows five singlet at 2.25, 5.89, 6.60, 13.09 and 13.66 ppm characteristic of methyl, NH_2_, H4 of pyrazole, NH and SH groups, respectively. The ^13^C NMR spectrum of **Pyz-2**, shows a signal at 10.69 ppm associated with the carbon of the CH3 group, a signal at 05.08 ppm characteristic of the tertiary carbon of the pyrazole, followed by two signals of the quaternary carbons of pyrazole ring, which appear at 138.64 and 139.90 ppm, and the two characteristic signals of carbons 1,2,4-triazole resonate at 155.40 and 165.06 ppm.

The mass spectra (ESI) show a peak related to the molecular ions at *m/z* = 260.2 and 197.0 thus confirming the proposed structures of **Pyz-1** and **Pyz-2**, respectively.

### Computational results

#### Electronic properties

**Figure 1:**
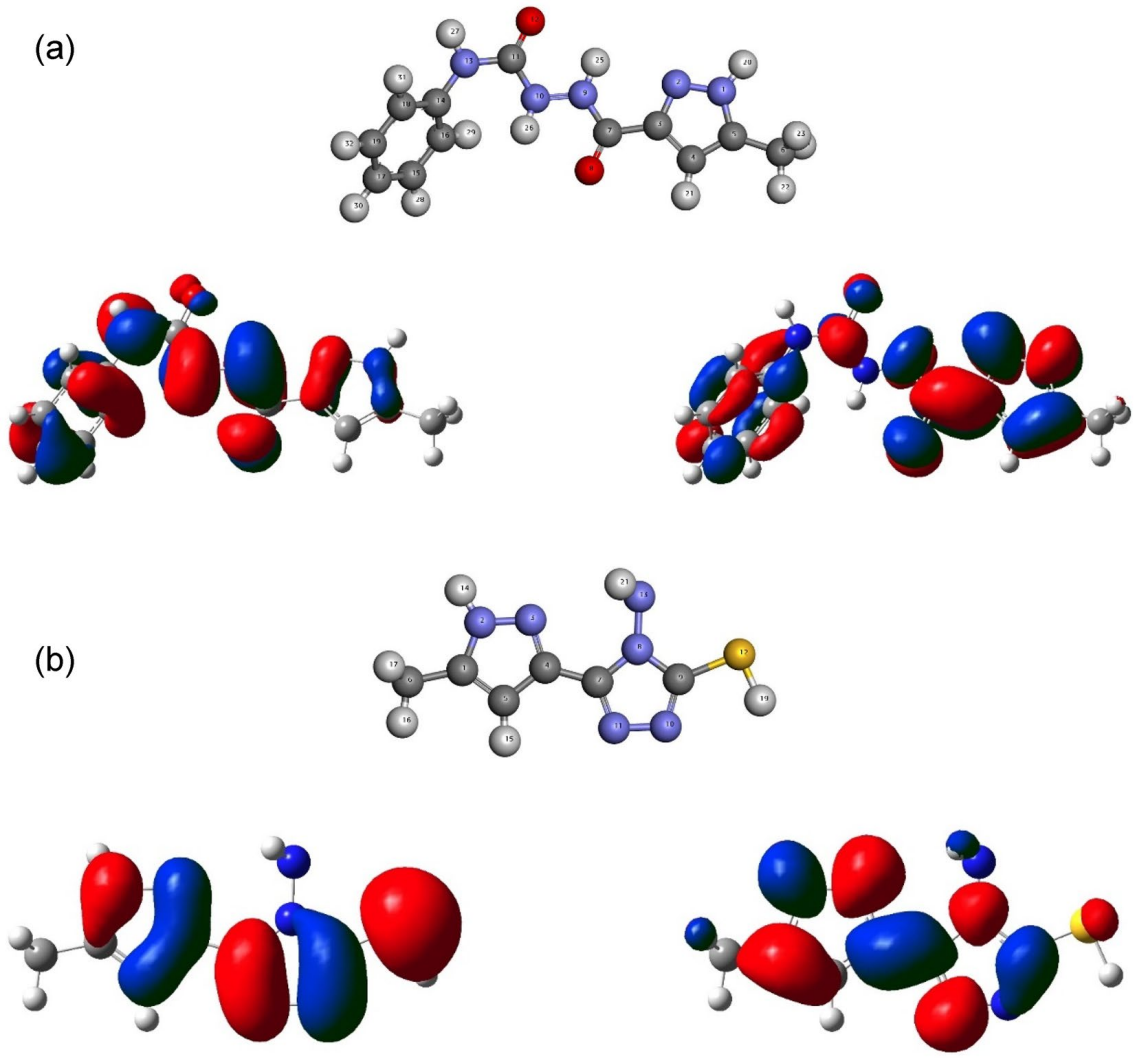
Optimized molecular structures and FMO’s density distributions of (**a**) **Pyz-1** and (**b**) **Pyz-2.**

Electronic and thermodynamic parameters are an effective way to explain the stability and reactivity of molecules. The physical quantities of **Pyz-1** and **Pyz-2** calculated at B3LYP/6–311G +  + (d,p) level of theory are given in Table 1, the optimized structures of the most stable conformer of **Pyz-1** and **Pyz-2** with the numbering of atoms are shown in Fig. 1.

**Table 1:** Ground state energies and thermodynamical parameters of **Pyz-1** and **Pyz-2**.

	Pyz-1		Pyz-2	
	Gas	Water	Gas	Water
Energy (au)	− 889.560233	− 889.580733	− 960.268510	− 960.287960
Polarizability (au)	193.402	254.749	139.329	184.588
Dipole Moment (D)	2.660	2.991	6.585	8.747
∆H (au)	− 889.292364	− 889.313353	− 960.101245	− 960.121676
∆G (au)	− 889.360095	− 889.381661	− 960.155981	− 960.173090
∆Ethermal (Kcal/mol)	167.498	167.191	104.368	103.752
Cv (cal/molK)	65.152	65.480	45.239	43.155
S (cal/molK)	142.552	143.766	115.203	108.210

The total energies of title molecules have been calculated for **Pyz-1** as − 889.560233 au in gas phase and − 889.580733 au in solvated phase and for **Pyz-2** as − 960.268510 au in gas phase and − 960.287960 au in solvated phase. The thermodynamic and kinetic preferability is reduced with the introduction of solvation resulting in higher stability in aqueous phase relative to the gas phase. The quantity of heat needed to increase a substance's temperature by one degree is referred to as the heat capacity, the Cv quantities of the compounds were computed as 65.152 cal/molK in gas phase and 65.480 cal/molK in water phase for **Pyz-1** and 45.239 cal/molK in gas phase and 43.155 cal/molK in water phase. The randomness of the system is quantitatively measured by the entropy. The computed values for **Pyz-1** and **Pyz-2** in gas and water phases are respectively 142.552 cal/molK, 143.766 cal/molK, and 115.203 cal/molK and 108.210 cal/molK. These findings are a major asset in the application of such structures to biological systems.

#### FMO’s analysis

Within computational chemistry, the energies and allocations of FMO’s are imperative reactivity descriptors. The highest occupied molecular orbital (HOMO) and the lowest unoccupied molecular orbital (LUMO) have significant roles in the electrical, electronic, optical properties, chemical reactivity and active sites of a given compound^51^. The HOMO's energy is proportional to the ionization potential (I) and describes the molecules sensitivity to electrophile attack^52^. The energy of LUMO is linked to the electron affinity (A) and defines the molecule's response to nucleophilic attack. A graphical illustration of the HOMO and LUMO for **Pyz-1** and **Pyz-2** is presented in Fig. 1. The positive phase is colored red, whereas the negative phase is colored blue. As shown in Fig. 1, with the exception of the methyl (CH_3_) groups, the highest HOMO and lowest LUMO orbitals of the **Pyz-1** and **Pyz-2** molecules are mainly localized almost throughout the structures.

The energy difference between the HOMO and LUMO orbitals determines the kinetic stability of the title compounds. Thus, molecules with a small energy gap are highly polarizable and are usually associated with high chemical reactivity as well as low kinetic stability^53^. According to the indications in the literature and the results reported in Table 2, a slight trend towards an increase in the energy gap can be observed when introducing the solvent effect for both titled molecules resulting in a lower reactivity in the aqueous phase. Furthermore, the energy gap reveals that **Pyz-1** (E_LUMO-HOMO_ = 5.118 eV) exhibits a lower value compared to **Pyz-2** (E_LUMO-HOMO_ = 5.166 eV), indicating easy molecular charge transfers, low kinetic stability and high chemical reactivity for **Pyz-1**.

**Table 2:** E_HOMO_, E_LUMO_ and ΔE_LUMO–HOMO_ energies at B3LYP/6–311G(d,p) level.

	Pyz-1		Pyz-2	
	Gas	Water	Gas	Water
E_HOMO_ (eV)	− 6.150	− 6.404	− 6.102	− 6.394
E_LUMO_ (eV)	− 1.032	− 1.269	− 0.937	− 1.043
∆E_LUMO-HOMO_ (eV)	5.118	5.136	5.166	5.352

#### Reactivity descriptors

Due to their wide range of applications in fields such as biology, chemistry and drug design, the development of chemical reactivity descriptors has gained considerable momentum^54^. Conceptual DFT theory is a developed branch of DFT which consists in deriving relevant concepts and principles from the electron density in order to make it possible to understand and predict the overall trends in chemical reactivity of a molecule^55^. With this aim, quantum chemical reactivity parameters have been calculated for the title compounds and their values are reported in Table 3.

**Table 3:** Quantum chemical reactivity parameters of **Pyz-1** and **Pyz-2** in eV.

Parameter	Pyz-1	Pyz-2
I	6.150	6.102
A	1.032	0.937
µ	− 3.591	− 3.520
η	5.118	5.165
ω	1.260	1.199
ω +	0.104	0.085
ω-	4.091	4.178
∆Nmax	0.702	0.681
∆Ec	− 1.260	− 1.199
∆Ec/∆Nmax	− 1795	− 1761

Global hardness (η) is related to the resistance to electronic transfer between a molecule and its environment. It represents the gap between the E_HOMO_ and E_LUMO_ orbital energies and is associated with the stability of chemical systems. Soft molecules exhibit a small energy gap and are more reactive than hard ones because of their ability to easily donate electrons to an acceptor. The calculations show that **Pyz-1** is less resistant to electronic transfer than **Pyz-2** and therefore would be more reactive. It should be noted that whenever the ratio of energy change to maximum charge acceptance ∆Ec/∆Nmax = 0, this indicates that the molecule under consideration is electron-saturated and has no predisposition for charge transfer^56^. According to the tabulated values in Table 3, the ∆E_c_/∆N_max_ values are − 1.795 eV and − 1.761 eV for **Pyz-1** and **Pyz-2**, respectively, with a maximum charge acceptance value of approximately 0.7 eV for both compounds. These values clarify the intramolecular charge transfer within the studied compounds and their ability to interact and bind to the active sites of α-glucosidase and α-amylase enzymes. Furthermore, the compounds' behavior suggests a tendency toward electrodonation rather than electroacceptance (ω^−^ > ω^+^).

#### Molecular electrostatic potential (MEP) map

**Figure 2:**
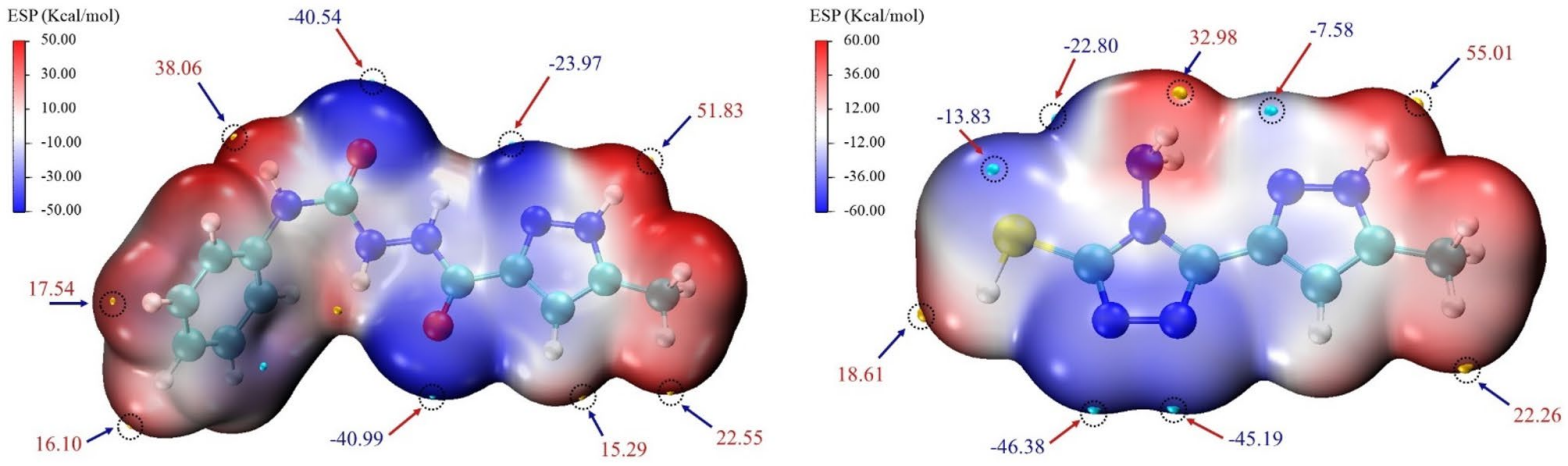
Computed molecular electrostatic potential surface (MEP) of Pyz-1 and Pyz-2.

Molecular electrostatic potential is a three-dimensional representation allowing the visualization of charge distributions, the prediction of reactivity towards electrophilic and nucleophilic attacks and the relative polarity of a given molecule^57^. Moreover, it is used in biological recognition processes and to evaluate hydrogen bonding interactions. The visualization of the MEP relies on the mapping of the values onto the area corresponding to the boundaries of the molecule using the BWR (BlueWhite-Red) color transition: red, blue and white representing the regions of most positive, most negative and neutral electrostatic potential respectively^58^. The 3D maps of the MEP of the investigated molecules are displayed in Fig. 2.

A high polarity is noted for **Pyz-1** and **Pyz-2** molecules. Indeed, we can notice that two distinct regions are formed: an electronically deficient region formed around the hydrogen atoms on the amine groups due to their high electronegativity, leading to a maximum value of 51.83 kcal/mol and 55.01 kcal/mol respectively for **Pyz-1** and **Pyz-2**. These observations reveal a strong tendency to attract negatively charged atoms electrostatically and to act as a hydrogen bond donor group. Furthermore, For **Pyz-1**, the overall minimum values on the surface are − 40.99 kcal/mol and − 40.54 kcal/mol due to the isolated pairs of ketone groups. Whereas, for **Pyz-2** the minimum values are − 46.38 kcal/mol and − 45.19 kcal/mol owing to isolated pairs of nitrogen atoms which are the most reactive sites for electrophilic attack during the interaction. From the MEP map, it can be derived that **Pyz-1** and **Pyz-2** molecules have significant biochemical activity and are key to drug recognition in biological systems.

#### NBO analysis

Natural bond orbital (NBO) analysis represents a computational tool for a more understandable and user-friendly interpretation of the computational solutions of the Schrödinger equation in chemical bonding concepts^59^. This approach allows studying the distribution of electron density on atoms and in bonds in an efficient way. It also provides a convenient way to examine charge transfer or conjugate interaction in molecular systems^60^. A useful aspect of the NBO method is that it provides information about the interactions of both filled (donor) orbital spaces (bonding orbitals or solitary pairs) and empty (anti-bonding and/or Rydberg orbitals) that could improve the analysis of intra-inter molecular interactions^61^. The strength of the interaction between electron donors and electron acceptors and thus the degree of conjugation of the system is measured by the value of the energy of hyperconjugal interactions, E(2). The larger this value, the stronger the interaction between electron donors and electron acceptors and the greater the degree of conjugation of the whole system.

To assess the donor–acceptor interactions, the second-order Fock matrix was performed. The stabilization energy E(2) associated with the i → j delocalization for each donor (i) and acceptor (j) is estimated as:

$$E_{2} = \Delta E_{ij} = \frac{{q_{i} \left( {F_{ij} } \right)^{2} }}{{\varepsilon_{j} - \varepsilon_{i} }}$$

where q_i_ is the donor orbital occupancy (2 for closed-shell, 1 for openshell), ε_i_, ε_j_ are diagonal elements (orbital energies), and F_ij_ is the off-diagonal NBO Fock matrix element.

For both compounds, the most important interaction analyses between the occupied Lewis-type NBO orbital (bonding) and the unoccupied non-Lewis-type NBO orbital (anti-bonding) have been calculated and donor–acceptor interactions having a stabilization energy above 20 kcal/mol are presented in Table S1 and S2. Inspection of different donors and acceptors reveals three types of donor’s LP, σ and π, and two types of acceptors σ* and π*. In **Pyz-2**, a remarkable stabilization energy contribution was noticed due to the interaction of filled molecular orbitals σ(N13-H21) and lone pairs LP(N13) with unfilled σ*(C7-N11) molecular orbital. The perturbative energy for these interactions are respectively 704.06 kcal/mol and 556.85 kcal/mol, which result in an important intramolecular charge transfer (ICT) causing a high stabilization of the investigated compound. The essential interaction energies related to SM3 molecule reveal that the most relevant donor–acceptor interactions are the interactions between the lone electron pair (LP) of the nitrogen atoms LP(N9) and LP(N1) to the anti-bonding acceptor π*(C7-O8), π*(C4-C5) and π*(N2-C3) orbitals. The interaction energies reported for the mentioned conjugative interactions are 61.25 kcal/mol, 37.87 kcal/mol and 31.35 kcal/mol respectively. The relatively strong electron donor donation from the π(C14-C16) → π*(C15-C17) (20.89 kcal/mol), π(C15-C17) → π*(C18-C19) (22.11 kcal/mol) and π(C18-C19) → π*(C14-C16) (21.34 kcal/mol) of the aromatic ring are identified. This phenomenon results from intramolecular hyperconjugative interactions between the π (C–C) and π*(C–C) orbitals, which lead to an ICT causing stabilization of the system.

### Biological activities

#### Antidiabetic activity

Compounds **Pyz-1** and **Pyz-2** were studied in vitro for their antidiabetic activities against α-glucosidase and α-amylase enzymes. Acarbose was used standard drug. The results of α-glucosidase and α-amylase inhibitory activity are summarized in Table 4. As shown in the Table 4, compound **1** showed the lowest activity against α-glucosidase and α-amylase with IC_50_ values of 279.0 and 500 µM, respectively. **Pyz-1** and **Pyz-2** exhibited good α-glucosidase and α-amylase inhibitory activities with IC_50_ values of 75.62 ± 0.56 and 95.85 ± 0.92 µM, and 120.2 ± 0.68, 119.3 ± 0.75 µM, respectively, compared to Acarbose (IC_50(α-glucosidase)_ = 72.58 ± 0.68 and IC_50(α-amylase)_ = 115.6 ± 0.574 µM). The obtained results showed a highly significant inhibition on α-amylase and α-glycosidase for the studied compounds. It was found that structural modification of carbohydrazide **1** resulted in increased enzymatic inhibition for compounds **Pyz-1** and **Pyz-2**.

**Table 4:** α-glucosidase and α-amylase inhibitory activities of 1, Pyz-1 and Pyz-2.

Compounds	IC_50_ (µM)^a^	
	α-Glucosidase	α-Amylase
**1**	279.0 ± 4.03	** > *500***
**Pyz-1**	75.62 ± 0.56	119.30 ± 0.75
**Pyz-2**	95.85 ± 0.92	120.20 ± 0.68
**Acarbose**	72.58 ± 0.68	115.60 ± 0.57

^a^Values represent mean ± standard deviation (n = 3).

#### Antioxidant Activity

The antioxidant properties of the target compounds were determined by using DPPH, ABTS, H_2_O_2_ free radical scavenging_,_ reducing power (FRAP), and Xanthine Oxidase (XO) assays. Ascorbic acid (A.A) and allopurinol were used as a standard. The results are summarized in Table 5.

**Table 5:** Antioxidant activities of **Pyz-1** and **Pyz-2**.

Compound	IC50 (µM)^a^				
	DPPH	ABTS	FRAP	H_2_O_2_	XO
**Pyz-1**	238.53 ± 1.12	79.77 ± 0.45	100.20 ± 0.79	31.49 ± 0.92	24.32 ± 0.78
**Pyz-2**	138.70 ± 1.45	20.54 ± 0.87	62.62 ± 0.49	24.82 ± 1.23	10.75 ± 0.54
**Ascorbic acid**	78.11 ± 0.68	22.49 ± 0.59	88.12 ± 0.23	7.45 ± 1.11	–
**Allopurinol**	–	–	–	–	14.41 ± 1.01

^a^Values represent mean ± standard deviation (n = 3).

For DPPH assay, the antioxidant activity of **Pyz-2** was the highest as its SC_50_ value was the lowest (138.70 ± 1.45 µM), followed by **Pyz-1** with SC_50_ value of 238.53 ± 1.12 µM. **Pyz-1** and **Pyz-2** exhibited moderate antioxidant capacities. For ABTS assay, compound **Pyz-2** exhibited the potent scavenging activity with SC_50_ value of 20.54 ± 0.87 µM, which is 3.88-fold more than that **Pyz-1** (SC_50_ = 79.77 ± 0.45 µM). **Pyz-2** has an antioxidant activity comparable to that of A.A (SC_50_ = 22.49 µM). For the reducing power ability (FRAP), **Pyz-2** displayed the maximum reducing power with IC_50_ value of 62.62 ± 0.49 µM. Statistically, the reducing power of **Pyz-2** was stronger than A.A (IC_50_ = 88.12 µM). Moreover, in H_2_O_2_ method, **Pyz-1** and **Pyz-2** displayed a good antioxidant activity with IC_50_ values of 31.49 ± 0.92 and 24.82 ± 1.23 μM, respectively, compared to A.A (IC_50_ = 7.45 µM).

The results inhibitory effect on xanthine oxidase of title compounds, showed that **Pyz-1** exhibited a considerable XO activity with IC_50_ value of 24.32 ± 0.78 µM. Furthermore, **Pyz-2** manifested the highest XO inhibitory activity with IC_50_ value of 10.75 ± 0.54 µM, compared to Allopurinol (IC_50_ = 14.41 ± 1.01 µM).

### Docking studies

**Figure 3:**
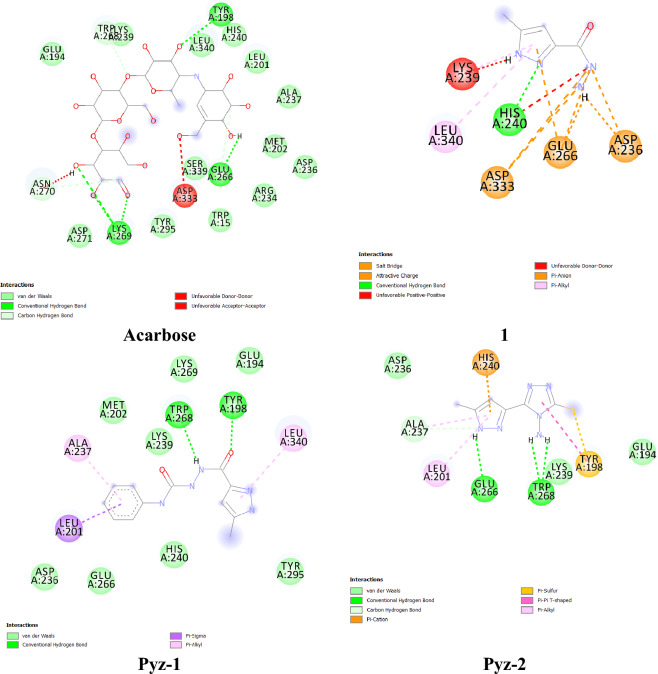
Binding between the docked compounds (**1**, **Pyz-1**, **Pyz-2** and Acarbose) and α-amylase (PDB = 2GJP).

**Figure 4:**
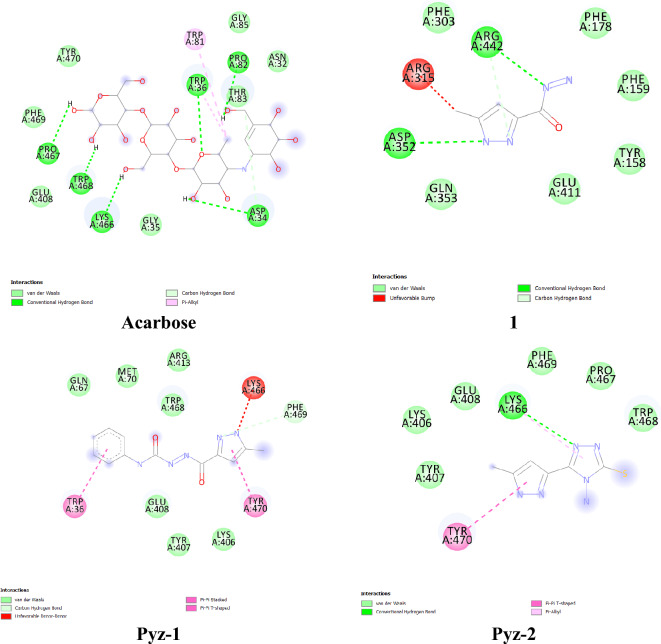
Binding between the docked compounds (**1**, **Pyz-1**, **Pyz-2** and Acarbose) and α-glucosidase (PDB:3A4A).

The binding interaction of the **1**, **Pyz-1** and **Pyz-2** ligands with α-amylase and α-glucosidase was further examined by performing an in silico binding interaction analysis to understand their interactions at the enzymes active site. Similarly, Acarbose (ACA), a standard type 2 antidiabetic drug, was docked as a positive control**.** The most probable docking positions of ligands exhibiting the best binding affinity towards targeted enzyme α-amylase and α-glucosidase are grouped in Table 6 and illustrated in Figs. 3 and 4.

**Table 6:** Docking results of the binding affinity and RMSD values of different poses in 3A4A and 2GJP.

Ligands	α-glucosidase (PDB = 3A4A)		α-amylase (PDB: 2GJP)	
	Affinity (Kcal/mol)	Rmsdl.b	Affinity (Kcal/mol)	Rmsdl.b
**1**	− 5.3	0.000	− 5.3	0.000
**Pyz-1**	− 5.7	0.000	− 6.7	0.000
**Pyz-2**	− 4.3	0.000	− 5.6	0.000
**ACA**	− 5.9	0.000	− 6.2	0.000

As noted from Table 6, all values of binding energies are negative which stabilize the systems and favor interactions between the proteins and the ligands.

#### *Against α-amylase enzyme target (PDB* = *2GJP)*

Among the newly suggested alternative compounds for treating diabetes, **Pyz-1** and **Pyz-2** demonstrate binding energies of − 6.7 kcal/mol and − 5.6 kcal/mol, respectively, both higher than the binding energy of ligand **1**, which stands at − 5.3 kcal/mol.

Figure 3 presented the interacted amino acid residues of α-amylase with **Pyz-1**. The surrounding amino acid residues of the enzyme included Tyr198, Trp268, Ala237, Leu201 and Leu340. Two hydrogen bonds are formed in **Pyz-1**-α-amylase complex between hydrogen atom of Tyr198 residue and lone pairs of oxygen atom of keto group and between lone pairs of oxygen atom of keto group of Trp268 residue and hydrogen atom of amin group of **Pyz-1** for a distance of 2.44 Å and 1.92 Å respectively. Furthermore, the π-sigma and π-Alkyl interactions between benzene ring and amino acids residues, consolidated the **Pyz-1** scaffold in the Pyz-1-α-amylase complex. **Pyz-2** molecule interacted α-amylase by establishing a conventional hydrogen bond with Glu266 (2.34 Å) and two hydrogen bonds with Trp268 (2.30 Å and 2.60 Å) (Fig. 3). This compound also exhibits π-cation, π-Sulfur, π-Alkyl and π-π interactions with Leu201, His240 and Tyr198. For acarbose, selected as a reference, the binding study revealed the involvement of fourteen amino acids in the molecular interactions, including five hydrogen bonds with the amino acids Lys269, Glu266 and Tyr198, two carbon hydrogen bonds with Asn270 and Tyr268 amino acids as well as eight Van der Waals type interactions with Asp271, Tyr295, Arg234, Ala237, His240, Leu340, Lys239 and Glu194 amino acids (Fig. 3). Unfavorable donor-donor and acceptor-acceptor interactions are also observed with the amino acid Asp333. Regarding compound **1**, the affinity with α-amylase resulted in a binding energy of − 5.3 kcal/mol.

#### *Against α-glucosidase enzyme target (PDB* = *3A4A)*

From the Table 6, the positive control ACA was found to be most effective α-glucosidase binder, thus forming the most stable complex with the lowest energy (− 5.9 kcal/mol) and interacting with Pro467, Trp468, Lys466, Asp34, Trp36 and Pro82 amino acids via strong hydrogen bonds (H-acceptor and H-donor). The second-most effective binder was **Pyz-1** with an energy value of − 5.3 kcal/mol. Seven Van der Waals interactions, two π-π interactions and a single carbon hydrogen bond stabilized the **Pyz-1**-α-glucosidase complex (Fig. 4). Thus, the residues realizing Van der Waals interactions were Gln67, Met70, Arg413, Trp468, Glu408, Tyr407 and Lys406. Furthermore, amino acid residues such as Trp36 and Tyr470 also made a contribution to the stability of the complex through π-π interactions (Fig. 4). In the less stable **Pyz-2**-α-glucosidase complex (− 4.3 kcal/mol), numerous intermolecular interactions were established between the docked pyrazole and the active α-glucosidase amino acids, including classical hydrogen bond, Van der Waals interactions, π-Alkyl and π–π interactions (Fig. 4). Concerning compound **1**, the binding energy with α-glucosidase was − 5.3 kcal/mol. In general, the binding energy analysis performed in this section for each coupling between the ligands (Acarbose, **1**, **Pyz-1** and **Pyz-2**) and the α-amylase and α-glucosidase macromolecule gives valuable information for predicting the affinity with the proteins. This analysis identifies which amino acid of proteins is in contact with the docked ligands in the active site.

### ADME-T analysis

In this study, a computational study of **Pyz-1** and **Pyz-2** was conducted to determine the surface area and other physicochemical properties according to the directions of Lipinski’s rule^62,63^. Lipinski suggested that the absorption of a compound is more likely to be better if the molecule achieve at least three out of four of the following rules: (i) HB donor groups ≤ 5; (ii) HB acceptor groups ≤ 10; (iii) M. Wt less than 500; (iv) logP less than 5. As shown in Table 7, the two compounds (Pyz-1 and Pyz-2) obey all Lipinski's rules. **Pyz-1** and **Pyz-2** have a number of hydrogen‐bonding acceptor groups 3 and 2 and only 4 and 3 hydrogen‐bonding donors, respectively. Also, molecular weights are less than 500 and logP less than 5 and all these values agree with Lipinski's rules.

**Table 7:** ADMET profile of **Pyz-1** and **Pyz-2*.***

Parameter	Pyz-1	Pyz-2
Molecular Weight (g/mol)	259.26	196.23
Lipophilicity (LogP)	1.044	0.64
H-bond donors	4	3
H-bond acceptors	3	2
Polar Surface Area (Psa)	98.91	88.31
Distribution At Ph 7.4 (Logd)	1.447	0.245
Plasma Protein Binding (Ppb)	29.32%	30.68%
Rat Acute Oral Toxicity	0.026	0.008
Caco-2 Permeability	− 5.303	0.023
Cyp1a2 Inhibitor	0.028	0.083
Cyp1a2 Substrate	0.334	0.054
Cyp2c19 Inhibitor	0.034	0.059
Cyp2c19 Substrate	0.052	0.038
Cyp2c9 Inhibitor	0.019	0.91
Cyp2c9 Substrate	0.908	0.001
Cyp2d6 Inhibitor	0.003	0.147
Cyp2d6 Substrate	0.282	0.012
Cyp3a4 Inhibitor	0.011	0.101
Cyp3a4 Substrat	0.045	0.023

Also, ADMET profiles of **Pyz-1** and **Pyz-2** were preliminary assessed to analyze their potentials to build up as good medication candidates^64–66^. As shown in Table 7, the initial logD values of **Pyz-1** and **Pyz-2** are 1.447 and 0.245, respectively, thereby suggesting that the distribution of the title compounds remains constant up to pH 6.0, and then starts to vary with the formation of charged species. On the other hand, the logP values of **Pyz-1** and **Pyz-2** are 1.044 and 0.64, respectively, suggest that the title compounds may be slightly permeable to the biological barriers of physiological system, thus still having lipophilicity considered ideal in the intestinal absorption phase. The PSA value of **Pyz-1** and **Pyz-2** estimated at 98.91 and 88.31, respectively, shows a low permeability to the different biological barriers, with a slight affinity for plasma proteins evaluated at 29.32% and 30.68%**.**

Looking at the calculated parameter values, we find that these values are within the recommended standard. Thus, we can conclude that the molecules of the title had characteristics of drugability and their application as a theoretical drug is safe.

## Conclusion

In conclusion, two novel pyrazole derivatives **Pyz-1** and **Pyz-2** were synthesized, characterized and evaluated for their antidiabetic and antioxidant activities. **Pyz-1** and **Pyz-2** were evaluated in vitro for their anti-diabetic, antioxidant activities. For anti-diabetic activity result, **Pyz-1** and **Pyz-2** showed a potent α-glucosidase and α-amylase inhibition. For antioxidant activity results, compound **Pyz-2** showed excellent activity that the title compounds have considerable antioxidant and radical scavenger abilities. In xanthine oxidase assay, **Pyz-1** and **Pyz-2** exhibited remarkable inhibitory ability with IC_50_ values 24.32 ± 0.78 and 10.75 ± 0.54 µM, respectively. Also, molecular structure optimization, electronic and thermodynamic properties of **Pyz-1** and **Pyz-2** through DFT calculations supported the antidiabetic and antioxidant activities of the title compounds. However, as docking results it was found to have higher activity of compound **Pyz-1** with a docking score of -5.7 kcal/mol against α-glucosidase and -6.7 kcal/mol against α-amylase. The predicted ADMET profiles of the title compounds were in line with Lipinski's rules and we can conclude that **Pyz-1** and **Pyz-2** had drugability characteristics. These promising results encourage further study of the antidiabetic and antioxidant activities of the title compounds by measuring their toxicity. Thus, advanced in vivo studies are in progress and will be published elsewhere.

### Supplementary Information

Supplementary Information.

## Data Availability

All data generated or analyzed during this study are included in this published article and its supplementary information files.
