# Supplementary material for: Synthesis, structural characterizations, in vitro biological evaluation and computational investigations of pyrazole derivatives as potential antidiabetic and antioxidant agents
Source: Sci Rep. 2024 Jan 15;14:1312. doi: 10.1038/s41598-024-51290-6 (PMC10789823; doi:10.1038/s41598-024-51290-6)
Supplement: Supplementary file 1 — Supplementary Information. [file 41598_2024_51290_MOESM1_ESM.docx]

**Supplementary Material To**

Synthesis, structural characterizations, *in vitro* biological evaluation and computational investigations of pyrazole derivatives as potential antidiabetic and antioxidant agents

Salma Mortada^1^, Khalid Karrouchi^2*^, El Hadki Hamza^3^, Afaf Oulmidi^4^, Mashooq Ahamd Bhat^5^, Hassane Mamad^6^, Youssra Aalilou^1^, Smaail Radi^7^, M’hammed Ansar^8^, Azlarab Masrar^6^, My El Abbes Faouzi^1^

*^1^ Laboratory of Pharmacology and Toxicology, Biopharmaceutical and Toxicological Analysis Research Team, Faculty of Medicine and Pharmacy, Mohammed V University in Rabat, Morocco;*

*^2^ Laboratory of Analytical Chemistry and Bromatology, Team of Formulation and Quality Control of Health Products, Faculty of Medicine and Pharmacy, Mohammed V University in Rabat, Morocco;*

*^3^ CERNE2D: Laboratory of Spectroscopy, Molecular Modelling, Materials, Nanomaterials, Water and Enviroment (LS3MN2E), Faculty of Sciences, University Mohammed V in Rabat, Morocco,*

*^4^ Institute of Condensed Matter and Nanosciences, Molecular Chemistry, Materials and Catalysis (IMCN/MOST), Université Catholique de Louvain, 1348 Louvain-la-Neuve, Belgium;*

*^5^ Department of Pharmaceutical Chemistry, College of Pharmacy, King Saud University, Riyadh 11451, Saudi Arabia. (mabhat@ksu.edu.sa)*

*^6^ Central laboratory of hematology, Ibn Sina Hospital, Faculty of Medicine and Pharmacy, Mohammed V University in Rabat, Morocco;*

*^7^ Laboratoire de Chimie Appliquée et Environnement (LCAE), Faculté des Sciences, Université Mohammed I, 60000 Oujda, Morocco.*

*^8^ Laboratory of Medicinal Chemistry, Faculty of Medicine and Pharmacy, Mohammed V University in Rabat, Morocco,*

***** Correspondence authors:

[khalid.karrouchi@um5s.net.ma](mailto:khalid.karrouchi@um5s.net.ma) (K.Karrouchi)

**Table of contents**

- - 1. Experimental section……………………………………………………………………………………..S3
    2. FT-IR spectra of **Pyz-1**…………………………………………………………………….....................S7
    3. FT-IR spectra of **Pyz-2**…..……………………………………………………………………………….S8
    4. ^1^H-NMR spectrum of **Pyz-1** …..……….………………………………………………………………..S9
    5. ^13^C-NMR spectrum of **Pyz-1** ……..…………………………………………………………………...S10
    6. ^1^H-NMR spectrum of **Pyz-2** …..……….………………………………………………………………S11
    7. ^13^C-NMR spectrum of **Pyz-2** ……..…………………………………………………………………...S12
    8. ESI-HRMS spectrum of **Pyz-1** ……………………………………………………………………….S13
    9. ESI-HRMS spectrum **Pyz-2** …………………………………………………………………………..S14

1. **Experimental section**

**Standards and reagents:**

ρ-Nitrophenyl-α-D-glucopyranoside (pNPG), α-glucosidase from Saccharomyces cerevisiae, α-amylase from Bacillus licheniformis, buffer Solution, Dimethylsulfoxide: organic polar solvent for solubilizing products, Sodium Carbonate: solution, acarbose. All other reagents and standards were of analytical reagent grade.

**Antidiabetic activity**

**α-glucosidase inhibition assay**

The α-glucosidase inhibitory activity was performed by using  (ρ-nitrophenyl-α-D-glucopyranoside) as a substrate according to the method described by (Kee et al., 2013),with minor modifications. The α-glucosidase method is based on the inhibition of the enzyme. α-glucosidase, which hydrolyses (4-Nitrophenyl-α-d-glucopyranoside) to α-d-glucopyranose and P-nitrophenol of yellow color. The target compound was dissolved in DMSO and all the evaluated samples were dissolved in tampon phosphate at a series of concentrations. A mixture of 150 µl of the sample and 100 µl of PBS (pH = 6.7) containing the α- glucosidase enzyme solution (0.1U/ml) was incubated at 37 ° C for 10 min, after incubation, 200µl of pNPG (1 mM) was added to the mixture. The mixtures were incubated at 37 °C for 30 min. Then 1 ml Na_2_CO_3_ (0.1 M) was added to stop the reaction. The absorbance was measured at 405 nm. The results were expressed in percentage of inhibition according to the following formula:

***Inhibition (%) =*** $\left( \frac{\boldsymbol{A}\mathbf{Control}\boldsymbol{-Assample}}{\boldsymbol{AControl}} \right)\boldsymbol{*}100$

Where **A_Control_** refers to the absorbance of control (enzyme and buffer),

**A_Sample_** refers to the absorbance of sample (enzyme and inhibitor).

Acarbose was used as a positive control to compare the obtained results. The same reaction mixture without α-glucosidase was used as negative control.

**α-amylase inhibition assay**

The α-amylase assay was performed by reacting various concentrations of compounds with α-amylase and starch solution by following the DNSA method with minor modifications (Hashim et al., 2013). The evaluated samples were dissolved in tampon phosphate with various concentrations. The sample solution (10μL) was mixed with 240μL (sodium phosphate buffer 0.02M pH6.9) containing α-amylase (240U/mL), and incubated at 37°C for 20 min. After pre-incubation, 250 µL of 1% starch solution were added to each tube and incubated for 15 min. 1ml of dinitrosalicylic acid was added to stop the reaction, then incubate the solution in a water bath at 90°C for 10 min. The mixture was diluted with 1 mL deionized water and the absorbance (Abs) was measured at 540 nm.

The IC_50_ values were determined by the percentage of inhibitions at different concentrations. Acarbose was used as a standard drug. (positive control).

### Antioxidant Activity

**Standards and reagents**

DPPH (2,2-diphenyl-1-picrylhydrazyl), ABTS (2,2′-Azinobis-(3-Ethylbenzthiazolin-6-Sulfonic Acid), H_2_O_2_) hydrogen peroxide and ascorbic acid were purchased from Sigma–Aldrich. All other reagents and standards were of analytical reagent grade.

**DPPH radical scavenging activity assay**

The DPPH radical activity assay was performed following (Huang et al., 2011) with minor modification. The method using the stable free radical 2,2-diphenyl-1-picrylhydrazyl (DPPH) is based upon the reduction of DPPH free radical. Different concentrations of the prepared compounds (500, 250, 125, 62.5, 31.25 µM) were tested. The DPPH solution was prepared by dissolving 3,9mg of DPPH in 50 mL of the methanol. Then, 50µL of each concentration was added to 1,2ml of methanol and 250µL of the prepared DPPH solution (0.02 mM). The reaction mixture incubated in the dark for 30 min. The control was prepared by adding 1,25 mL of methanol to 250 µL of DPPH. Ascorbic acid was used as the standard. The absorbances of solutions were spectrophotometrically examined at 517 nm. The radical activity is expressed as inhibition ratio of initial concentration of DPPH radical and is calculated according to the formula:

**DPPH(%) =[(Abs_DPPH_ – Abs_Sample_)/Abs_DPPH_] * 100**

Where Abs_DPPH_ is the absorbance of DPPH radical and Abs_sample_ is the absorbance in the presence of sample. The activity result in this assay was expressed as IC_50_, which represents the concentration of the sample required to inhibit 50% of the free radical activity.

**ABTS assay:**

The ABTS assay was performed as previously described by (Tuberoso et al., 2013). ABTS+ radical was generated by oxidation of ABTS with potassium persulfate. The blue-green ABTS was produced through the reaction between 2 mM ABTS and 70 mM potassium persulfate in water. The mixture was left to stand in the dark for 12–16 h before use. The ABTS+ solution as diluted with methanol to an absorbance of 0.700 ± 0.005 at 734 nm. Then 2 mL of diluted ABTS solution were mixed with 100 μL of samples and absorbance was measured after 1 min incubation at room temperature. A standard curve was obtained by using Ascorbic acid as standard solution. The results were expressed as IC_50_, which represents the concentration of the sample required to inhibit 50% of the free radical activity.

**Ferric reducing power assay (FRAP):**

The ferric ion (Fe^3+^) reducing power assay was performed as previously described by (Amarowicz et al., 2004) with minor modifications. Briefly, 1 mL of the samples were mixed with 2.5 mL of 0.2 M sodium phosphate buffer (pH 6.6) and 2.5 mL of 1% potassium ferricyanide. The mixtures were incubated in a boiling water bath at 50 °C
for 20 min. Then, 2.5 mL of 10% trichloroacetic acid was added and centrifuged at 3000 rpm for 10 min. Finally, 2.5 mL of the supernatant were mixed with 2.5 mL distilled water and 0.5 mL FeCl_3_ solution (0.1%, w/v). The absorbance was measured at 700 nm and the results were expressed as IC_50_.

**Hydrogen Peroxide Activity H_2_O_2_:**

The hydrogen-donating activity, measured utilizing hydrogen peroxide radicals as the hydrogen acceptor was performed as previously described by (Muruhan et al., 2013) with minor modifications. Briefly a solution of hydrogen peroxide (40 mM) was prepared in phosphate buffer (pH 7.4). Different concentrations of the prepared compounds (62.5, 31.25, 15.62, 7.81 and 3.9 μM) were added to a hydrogen peroxide solution (0.6 mL, 40 mM). The absorbance of hydrogen peroxide at 230 nm was determined after 10 min against a blank solution containing phosphate buffer without hydrogen peroxide (or ascorbic acid as the control). The hydrogen peroxide percentage activity was then calculated using the following equation:

$$\boldsymbol{H}\boldsymbol{2}\boldsymbol{O}\boldsymbol{2\%=Ab'-}\frac{\boldsymbol{Ab}}{\boldsymbol{Ab'}}\boldsymbol{\times100}$$

Where *Ab’* is the absorbance of the control reaction and *Ab* is the absorbance in the presence of the samples.

**Reference**

Amarowicz, R., Pegg, R.B., Rahimi-Moghaddam, P., Barl, B., Weil, J.A., 2004. Free-radical scavenging capacity and antioxidant activity of selected plant species from the Canadian prairies. Food Chemistry 84, 551–562. https://doi.org/10.1016/S0308-8146(03)00278-4

Hashim, A., Khan, M. Salman, Khan, Mohd. Sajid, Baig, Mohd.H., Ahmad, S., 2013. Antioxidant and *α* -Amylase Inhibitory Property of *Phyllanthus virgatus* L.: An *In Vitro* and Molecular Interaction Study. BioMed Research International 2013, 1–12. https://doi.org/10.1155/2013/729393

Huang, B., Ke, H., He, J., Ban, X., Zeng, H., Wang, Y., 2011. Extracts of Halenia elliptica exhibit antioxidant properties in vitro and in vivo. Food and Chemical Toxicology 49, 185–190. https://doi.org/10.1016/j.fct.2010.10.015

Kee, K.T., Koh, M., Oong, L.X., Ng, K., 2013. Screening culinary herbs for antioxidant and α-glucosidase inhibitory activities. Int J Food Sci Technol 48, 1884–1891. https://doi.org/10.1111/ijfs.12166

Muruhan, S., Selvaraj, S., Viswanathan, P.K., 2013. In vitro antioxidant activities of Solanum surattense leaf extract. Asian Pacific Journal of Tropical Biomedicine 3, 28–34. https://doi.org/10.1016/S2221-1691(13)60019-2

Tuberoso, C.I.G., Boban, M., Bifulco, E., Budimir, D., Pirisi, F.M., 2013. Antioxidant capacity and vasodilatory properties of Mediterranean food: The case of Cannonau wine, myrtle berries liqueur and strawberry-tree honey. Food Chemistry 140, 686–691. https://doi.org/10.1016/j.foodchem.2012.09.071

**2. FT-IR spectra**


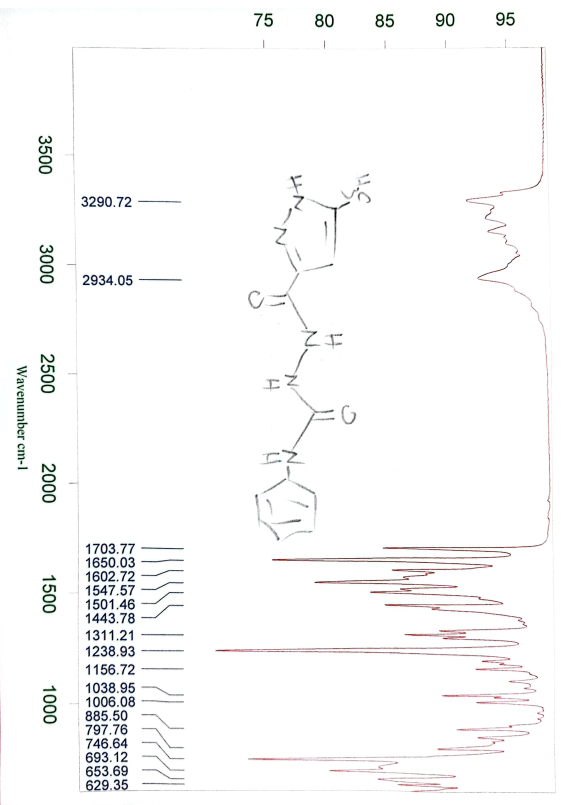


**Figure S1.** FT-IR spectrum of **Pyz-1.**


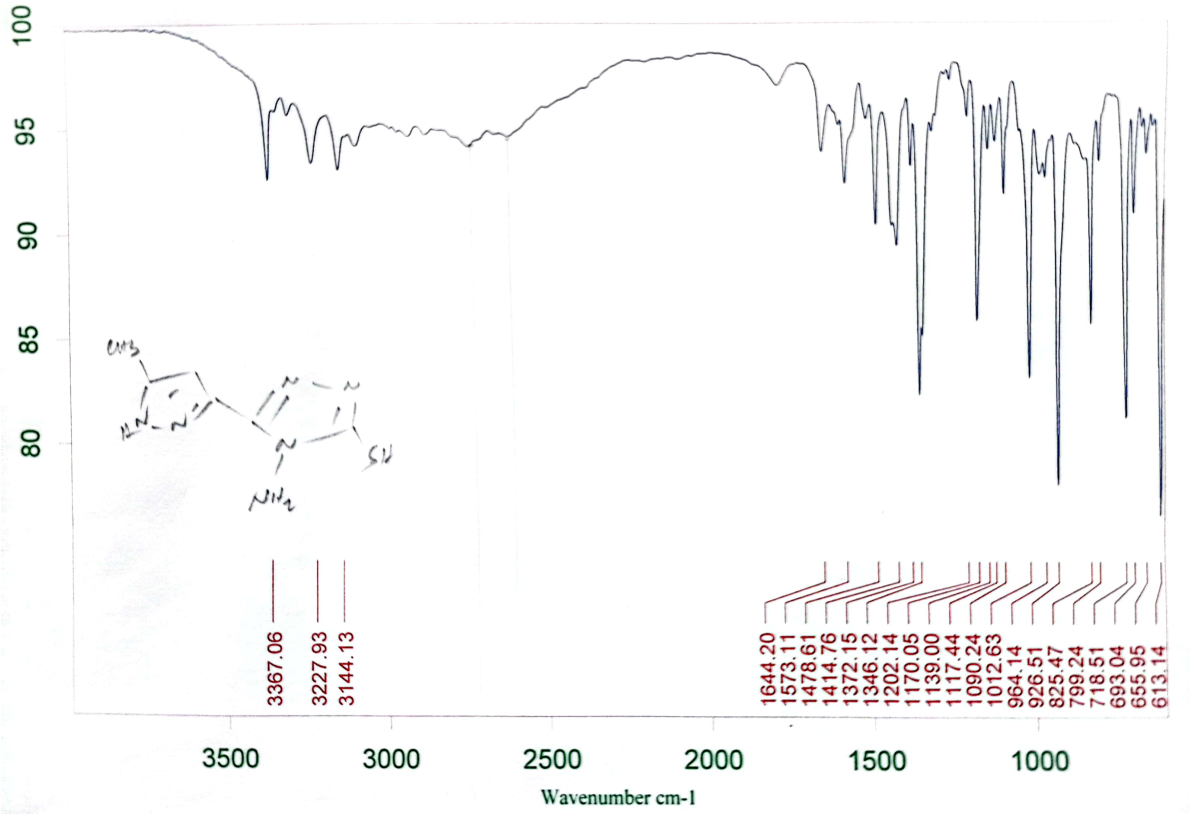


**Figure S2.** FT-IR spectrum of **Pyz-2.**

**3. NMR spectra**

**
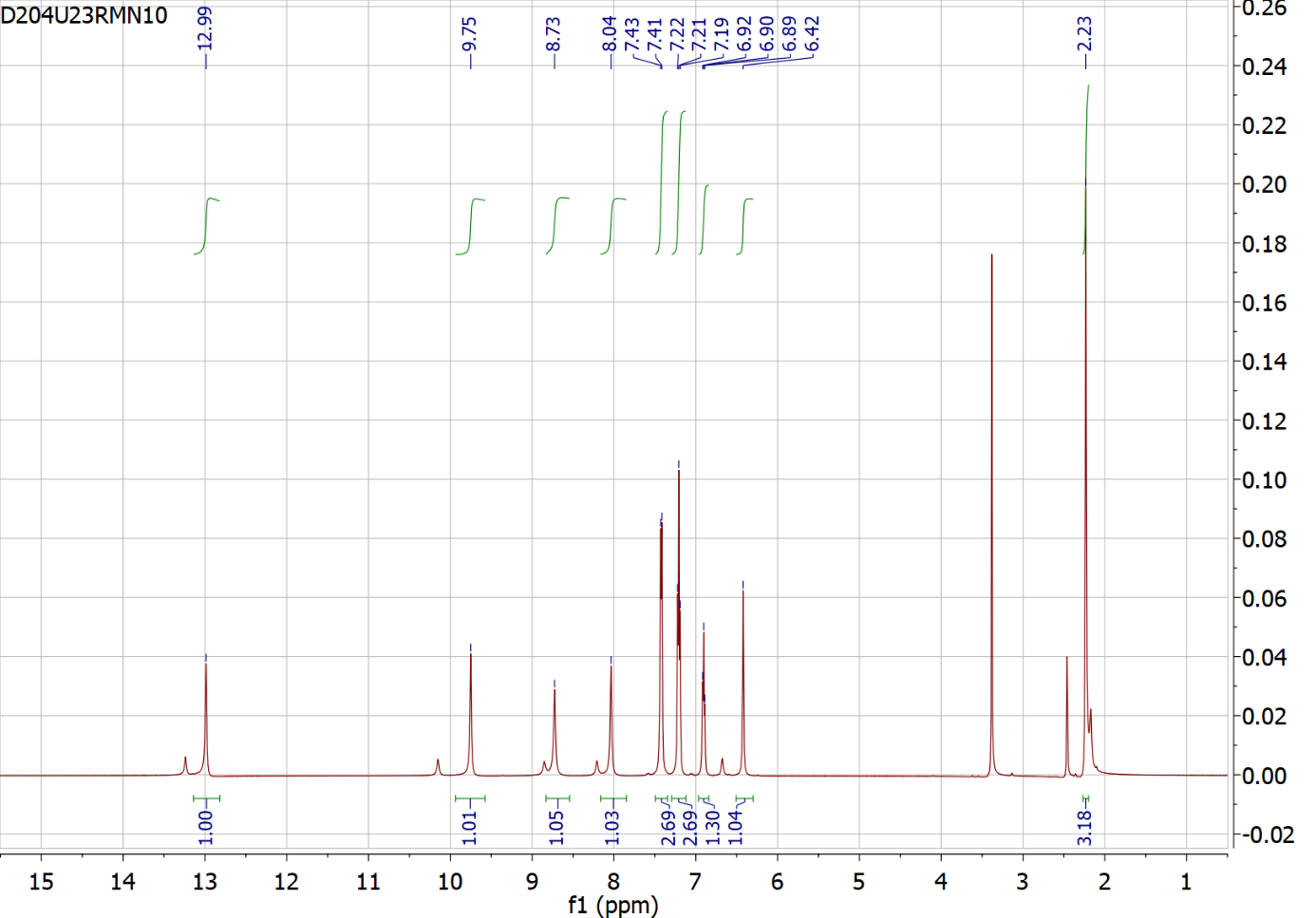
**

**Figure S3.** ^1^H NMR spectrum of **Pyz-1.**

**
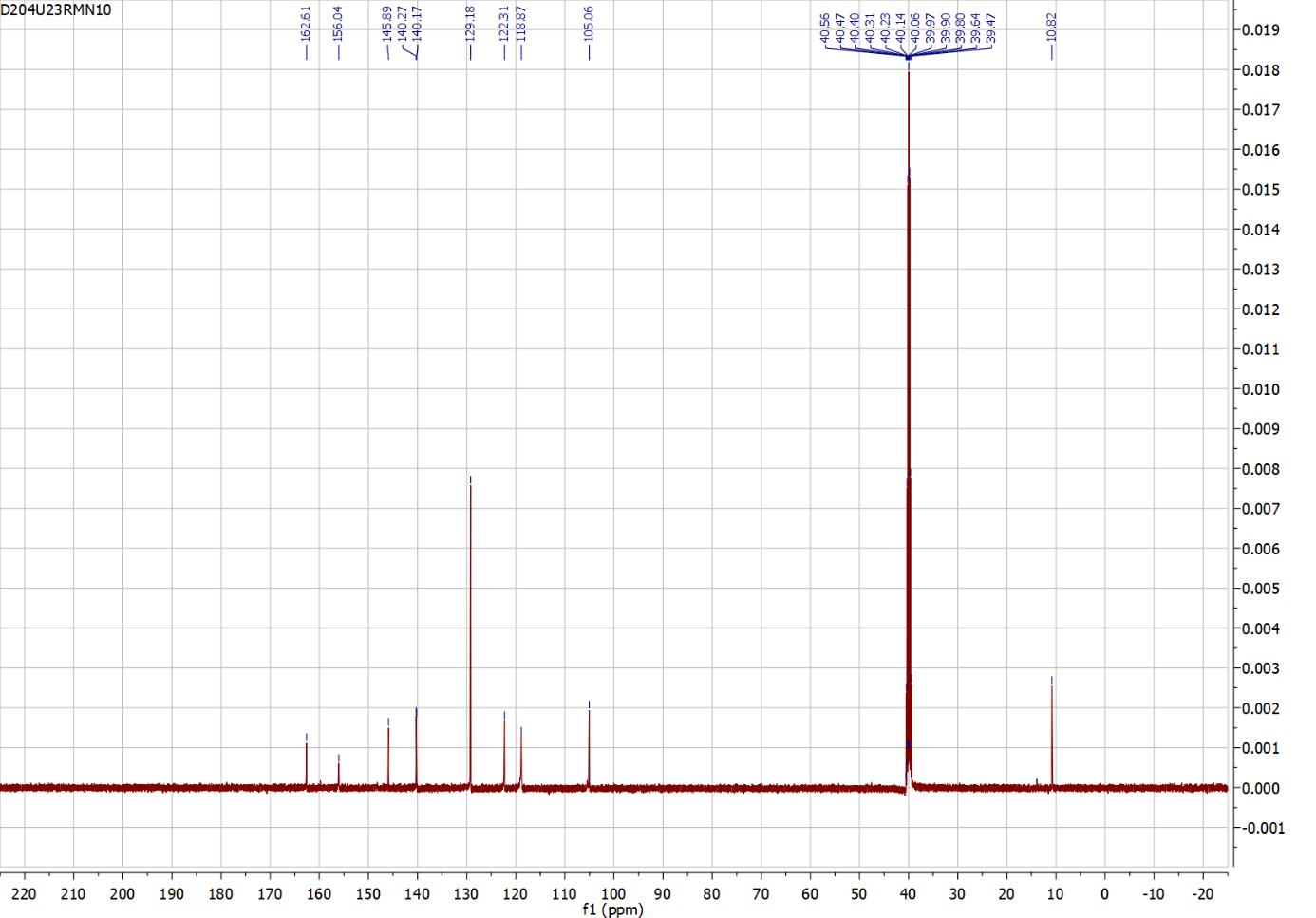
**

**Figure S4.** ^13^C NMR spectrum of **Pyz-1.**

**
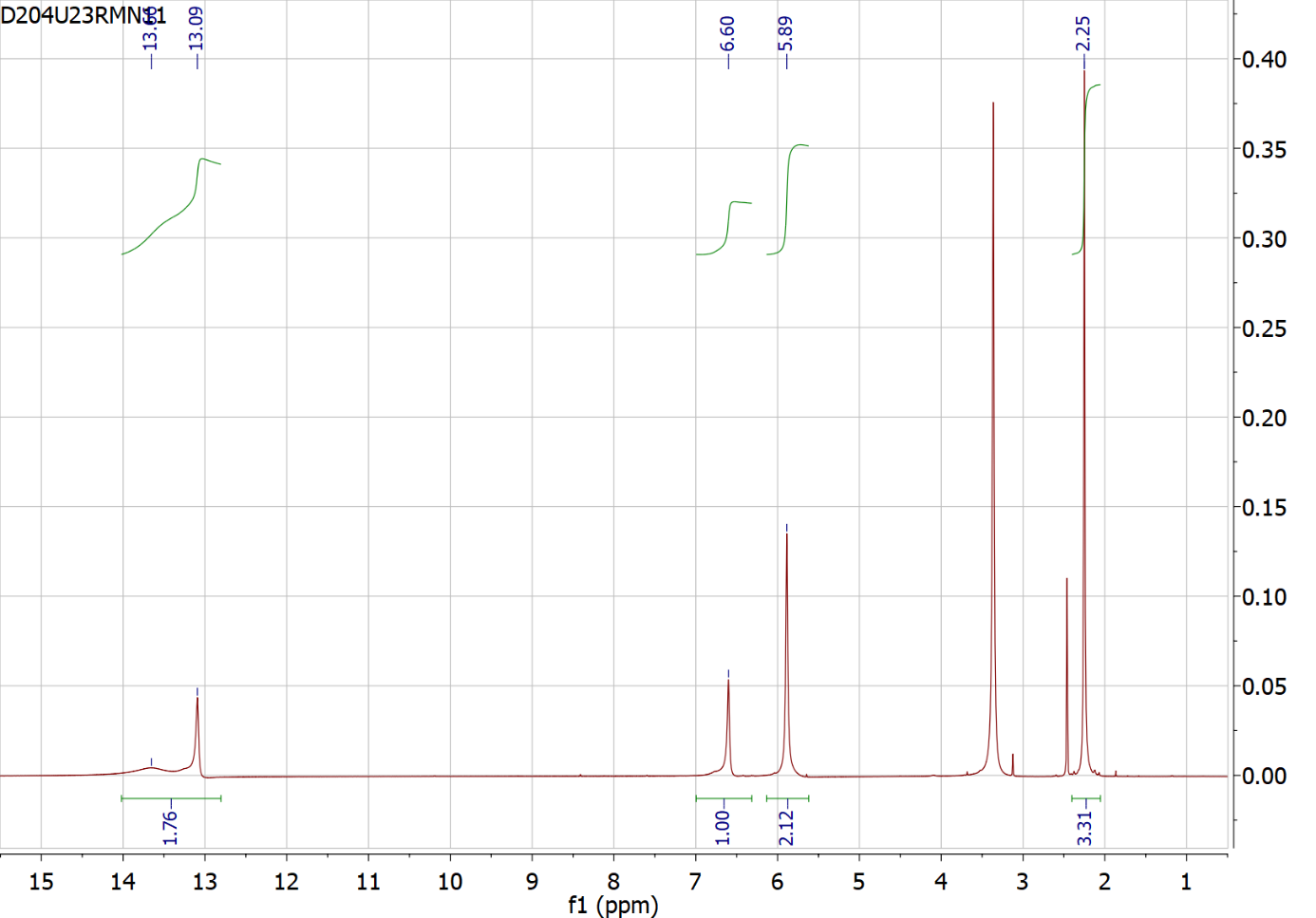
**

**Figure S5.** ^1^H NMR spectrum of **Pyz-2.**

**
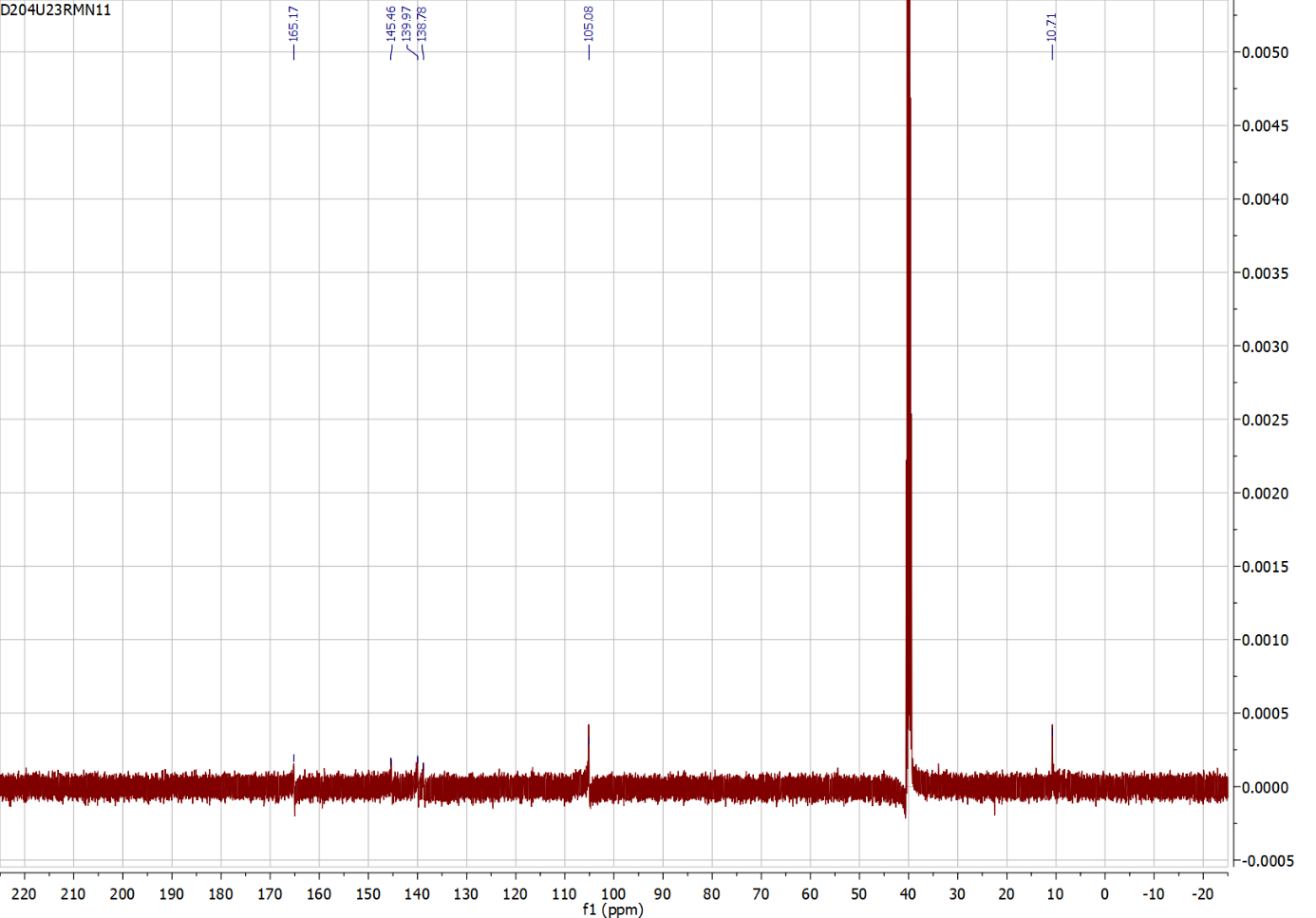
**

**Figure S6.** ^13^C NMR spectrum of **Pyz-2.**

**4. HRMS-ESI spectra**

**Figure S7.** ESI-HRMS spectrum of **Pyz-1.**

**Figure S8.** ESI-MS spectrum of **Pyz-2.**
